# Compact Digital Immunoassay Platform Integrating ELISA with a Lateral Flow Strip

**DOI:** 10.3390/biomedicines12112517

**Published:** 2024-11-04

**Authors:** Takuma Degawa, Yuma Hori, Masato Orikasa, Haruka Narita, Tomotaka Komori, Toru Yoshimura

**Affiliations:** Research and Development, Abbott Japan LLC, Matsudo 270-2214, Japan; takuma.degawa@abbott.com (T.D.); yuma.hori@abbott.com (Y.H.); haruka.narita@abbott.com (H.N.); tomotaka.komori@abbott.com (T.K.)

**Keywords:** lateral flow strip, Point-of-Care-Testing, digital immunoassay, digital ELISA, SARS-CoV-2

## Abstract

Background/Objectives: On-site diagnosis of infection in their early stages requires assays with high sensitivities that are compact and easy to operate out of the laboratory and hospital environments. However, current assay technologies fall short of these requirements and require highly skilled technicians to set up, operate, and interpret the results. Methods: To address these challenges, we developed and evaluated a Point-of-Care-Testing (PoCT) immunoassay platform called the D-strip. The D-strip platform combines the capabilities of a digital enzyme-linked immunoassay (ELISA) with a lateral flow assay (LFA). The D-strip sample flow cell is composed of the same components found in conventional LFAs, and its high sensitivity is due to its efficient implementation of ELISA. The fully integrated platform is simple and requires minimal user intervention to operate. Results: The D-strip exhibited a sample-to-result time of 15 min with a limit of detection (LOD) of 1.7 × 10^3^ copies/mL for severe acute respiratory syndrome coronavirus 2 (SARS-2-CoV) antigen. The LOD of the D-strip is 488-fold higher than that for conventional LFAs and is comparable to a clinical laboratory test. Conclusions: The D-strip is a compact and highly sensitive immunoassay platform with a strong potential for application as a confirmatory assay outside the clinical laboratory.

## 1. Introduction

The highly sensitive lateral flow assay (LFA) is a promising platform for early-stage disease diagnosis outside of the clinical laboratory setting. The global pandemic caused by coronavirus disease 2019 (COVID-19) revealed that conventional LFAs were unable to detect infected patients in the early stages of infection due to their low analytical sensitivity. Consequently, there is a need to develop highly sensitive and inexpensive point-of-care testing (PoCT) devices based on LFAs. In addition to the low analytical sensitivity of LFAs, the low sample yield collected outside hospital environments further reduces their accuracy. Minimally invasive, on-site specimen collection that does not require highly skilled technicians or sophisticated facilities remains a critical goal. To address these challenges, we aimed to develop a diagnostic platform that is highly sensitive, simple to use, and low-cost and that can be adapted for rapid testing of severe acute respiratory syndrome coronavirus 2 (SARS-CoV-2).

The sensitivity of LFAs can be enhanced by applying the following strategies: in situ signal amplification [1,2], sample pre-concentration [3,4], flow management [5,6], and the use of bright fluorescence reporters [7,8]. Compared to conventional immunoassays, digital immunoassays are ultrasensitive [9,10,11,12,13,14] and can analyze specimens with much smaller volumes [15]. However, commercially available digital immunoassay systems have large footprints and high operating power requirements [16]. Therefore, digital immunoassays are still not suitable for PoCT. Although several PoCT-oriented instruments for digital immunoassays have been proposed [11,15], a portable platform with a simple workflow remains to be developed. Molecular testing with nucleic acid amplification continues to be used for infectious diseases due to its high sensitivity and ability to prevent false-negative results. Relatively compact instruments for molecular tests have been launched on the market, such as Cobas^®^ Liat^®^ (Roche Diagnostics), based on polymerase chain reactions (PCR), and ID Now^®^ (Abbott Laboratories), based on nicking enzyme amplification reactions (NEAR). However, the reagents for molecular testing are generally more expensive compared with those for LFA. Therefore, the development of low-cost and highly sensitive LFAs remains necessary.

Here, to combine the simplicity and affordability of LFAs with advanced diagnostic capabilities, we present a compact and portable system that integrates a digital immunoassay (IA) with LFA, termed the “D-strip”. Digital IA, an ultrahigh-sensitivity immunoassay, utilizes encapsulated small solution-in-oil-sealed wells (femtoliter chambers) and fluorescent substrates. The small volume of the femtoliter chambers allows for rapid accumulation of fluorescent reaction products, generating detectable fluorescence signals in a short time. In contrast, traditional analog assays perform enzyme reactions in larger bulk volumes, which limits the detection of signals from small amounts of enzymes within reasonable timeframes. As a result, the sensitivity of digital IA is 1000 times higher than conventional ELISA methods [17]. Indeed, we have previously demonstrated ultrahigh sensitivity using digital IA [11,13]. The D-strip is highly sensitive to the SARS-CoV-2 nucleocapsid (N) protein, with a sample-to-result time of less than 15 min, comparable to clinical laboratory tests. To integrate digital IA into LFA, we simplified the digital IA process to preserve LFA’s inherent simplicity, in contrast to our previous benchtop system [11,13]. First, to reduce the footprint and power consumption of the D-strip system, we eliminated sample pipetting by using capillary-driven flow on the strip, which also enabled reagent mixing and antigen-antibody reactions without the need for complex mechanical components typically seen in conventional LFAs. Additionally, we incorporated a fixed magnet in the D-strip instrument to load magnetic beads into microwells, reducing the number of actuators required for magnet sliding, as seen in our earlier benchtop system. Furthermore, the assay workflow is also essentially the same as that for LFAs. The ultrahigh sensitivity of digital immunoassays relies on the enzymatic amplification of the fluorescence signal in a small-volume reactor. Fluorinated oil is generally used to partition the enzyme-conjugated antibody into oil-sealed microwells [11,13]. This requires complex handling mechanisms to replace the aqueous solution with oil. Although several alternatives have been proposed, including physical exclusion [17,18,19] and active airflow [20], additional processes for encapsulation were still required. In the D-strip assay, we discovered that passively wicking the solution with absorbent was sufficient to isolate the microwells from each other, meaning that no additional liquid handling or mechanical processes were required. These enhancements minimized user intervention, such as the manual injection of sample solution and chase buffer commonly seen in conventional LFAs. Furthermore, the imaging system is composed of a battery-powered compact camera and an LED excitation system. Therefore, the size of the D-strip platform is comparable to smartphone-based devices for digital assays [21,22,23]. As discussed in detail in the following sections, the D-strip provides highly sensitive, portable, and user-friendly immunoassays for PoCT.

## 2. Materials and Methods

### 2.1. Reagent

Inactivated SARS-CoV-2 was generously provided by the Japanese National Institute of Infectious Diseases. A virus suspension containing 10^6^ copies/mL in PBS-T, including 0.5% (*w*/*v*) bovine serum albumin, was diluted in a sample buffer containing 30.5 mM Tris Amino (MilliporeSigma, Burlington, MA, USA), 19.5 mM Tris HCl (MilliporeSigma, Burlington, MA, USA), and 0.011% (*w*/*v*) Poly-L-Lysin HBr (MilliporeSigma, Burlington, MA, USA) before use. Antibody-coated superparamagnetic particles (Ab-mP) and alkaline phosphatase-labeled antibodies (AP-labeled Ab) to SARS-CoV-2 N protein [24] and their proprietary diluents are described in a previous report [11]. Briefly, two types of antibodies against the SARS-CoV-2 N protein were developed at Yokohama City University (Kanto Chemical, Tokyo, Japan). One antibody was coated onto magnetic microparticles (Magnosphere MS300 carboxyl beads, JSR, Tokyo, Japan) using 1-ethyl-3-(3-dimethylaminopropyl) carbodiimide (Sigma-Aldrich, Burlington, MA, USA) in MES buffer. The other antibody for the N protein was conjugated to calf intestine alkaline phosphatase (BBI Solutions, Crumlin, UK) via click chemistry using trans-cyclooctene and tetrazine (Click Chemistry Tools, Scottsdale, AZ, USA) and purified with a Superdex 200 Increase column (Cytiva, Marlborough, MA, USA). The activity of the antibodies, as well as their lack of cross-reactivity with related human coronaviruses and other respiratory viruses, was confirmed in a previous report [11]. The chase buffer consisted of 290 mM pyranine–phosphate (Fujifilm Wako Pure Chemical, Osaka, Japan), an alkaline phosphatase substrate, 250 mM Tris Amino (MilliporeSigma), 0.5% (*v*/*v*) Tween-20 (Fujifilm Wako Pure Chemical, Osaka, Japan), 1.0 mM MgCl_2_ (Kanto Chemical, Tokyo, Japan), 2.5 mM Levamisole HCl (Tokyo Chemical Industry, Tokyo, Japan), and 0.15% (*w*/*v*) ProClin 950 (MilliporeSigma, Burlington, MA, USA).

The dried particles and conjugate pads were prepared as follows. A glass fiber pad (Merk, Rahway, NJ, USA, GFDX) supported by a scaffold was soaked in Ab-mP or AP-labeled Ab reagent (0.5 mL/mm^2^). After soaking, the pad was dried in a vacuum dryer (TAITEC, Saitama, Japan, VD-800R) at room temperature. To prevent nonspecific binding of the AP-labeled antibody to Ab-mP during long-term storage, we used separate pads for the Ab-mP and the AP-labeled antibody. The dried reagent pad was stored at <10% relative humidity before use.

### 2.2. Assembling the D-Strip Flow Cell

**Figure 1:**
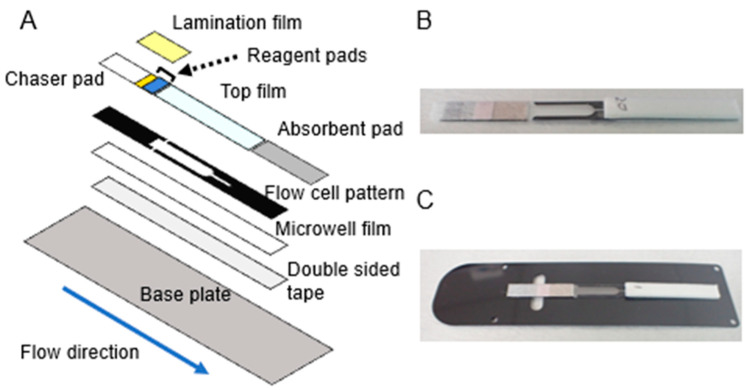
(**A**) Schematic of the D-strip flow cell fabrication. The chaser pad and reagent pads (yellow: conjugation pad and blue: microparticle pad), a surfactant-coated polyester top film, and an absorbent pad are aligned over the flow cell-patterned 100 µm thick sticky polyester film. All of the chaser pads, reagent pads, and absorbent pads are on the same level on flow cell-patterned polyester film. A part of the chaser pad and reagent pads are also covered with a polypropylene lamination film. The assembled pads and the flow cell pattern are attached to the microwell-patterned 0.188 mm thick COP film, forming the D-strip flow cell. The assembled flow cell is finally secured to a base film via double-sided tape. Images are not to scale. (**B**) The partially completed D-strip assembled following the scheme described in (**A**) before fixation to the base film. (**C**) The fully assembled D-strip.

The structure and representative images of the D-strip flow cell are shown in Figure 1. Double-sided tape 100 µm thick was custom manufactured (CSTEC corporation, Kyoto, Japan) from 50 µmthick black polyethylene terephthalate (PET) film (TORAY, Tokyo, Japan, Lumirror^®^ #50-X30) and 25 µmthick glue (3M, Alexandria, MN, USA, 9969) and shaped by a laser cutter to the form shown in Figure 1A. The flow cell outlet was coated with 1% (*v*/*v*) Tween-20 (Fujifilm Wako Pure Chemical, Osaka, Japan) and dried in ambient conditions for 10–15 min before lamination. Double-sided tape was stacked on a film-formed microwell array, which was manufactured by Sumitomo-Bakelite (Tokyo, Japan) from cyclo-olefin polymer (COP), coupling the COP backing with the other materials: chaser pad (Merk, Rahway, NJ, USA, GFDX), reagent-coated pads, hydrophilic transparent film (3M, Alexandria, MN, USA, 9901P) and absorbent (Cytiva, Marlborough, MA, USA, CF7). All pads were laminated using a single-side polypropylene tape (3M, Alexandria, MN, USA, 9793R) while keeping a small section of the sample pad bare for the sample application, as shown in Figure 1. The assembled serial strip was divided into single strips using a die cutter powered by a hand press machine (Road planning, Gunma, Japan, RV-2000). The single strip (Figure 1B) was adhered onto a 250 µmthick black PET film baseplate (custom fabricated by TORAY, Tokyo, Japan using Lumirror^®^ X30#250 by CSTEC, Kyoto, Japan) via double-sided tape (3M, Alexandria, MN, USA, 9965) (Figure 1C).

### 2.3. Assay Platform Design

**Figure 2:**
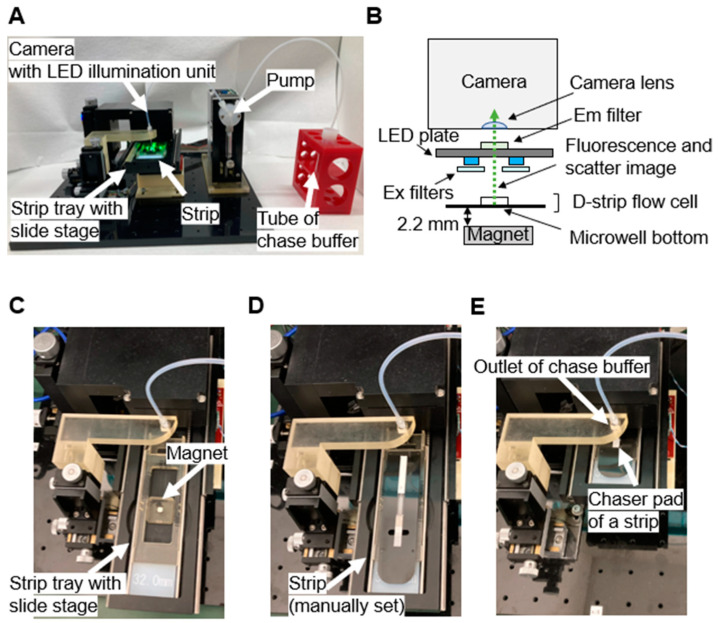
Assay and imaging system for the D-strip assay: (**A**) Photo of the system. D-strip’s system mainly contains a camera system with an LED illumination unit, stirp tray, and pump to perform programmed injection of chase buffer onto the strip after a given time during an assay. (**B**) Schematic of the optical system, D-strip flow cell placement, and magnet alignment. The D-strip flow cell is secured on a tray. The camera is focused on the microwells of the D-strip flow cell, with the LED plate (blue and green) illuminating the flow cell. The fluorescent solution in the microwell is excited by the blue LED through the excitation (Ex) filter, while the beads in the microwell are illuminated by the unfiltered green LED. The fluorescence emission and scattering images from the microwell are directed to the camera lens through the top film of the D-strip and the emission (Em) filter. The schematic is not to scale. (**C**–**E**) Photos of the system with an open, empty strip tray (**C**), an open strip tray loaded with a strip (**D**), and a closed strip tray loaded with a strip (**E**). A neodymium magnet was placed on the strip tray to load beads into the microwells during the D-strip assay.

The assay imaging system is composed of a consumer camera (Tough TG-6, OM Digital Solutions, Tokyo, Japan), syringe pump (Tecan, Mannedorf, Switzerland, Cavro^®^ XCalibur), linear stage (Thorlabs, Newton, NJ, USA, ELL17), and light-emitting diodes (LEDs) for sample excitation controlled by an Arduino Micro (Arduino, Boston, MA, USA). The consumer camera was operated using custom firmware, and the LEDs and optical filters were installed as described in a previous report [11]. In detail, four green LEDs (CREE, Durham, NC, USA, MLEGRN-A1-0000-000001) and four blue LEDs (CREE, Durham, NC, USA, XQEROY-H0-0000-000000N01) were soldered onto the illumination board in a quadrupolar arrangement. Each blue LED was covered with an excitation filter (Asahi Spectra, Tokyo, Japan, SV0490), and an emission filter (Asahi Spectra, Tokyo, Japan, LV0510) was mounted over the board aperture. All devices in the system were integrated using purpose-built software. The camera was mounted over the flow cell and collected images at a scale of 1.62 µm/pixel upon full zoom. A 4 mm × 4 mm × 4 mm (L × W × H) neodymium magnet (Magfine, Saitama, Japan, NS0025) was placed under the stage. A gap of 2.2 mm between the magnet and the bottom of the flow cell was maintained. A photo and schematic of the assay platform are shown in Figure 2, respectively.

### 2.4. D-Strip Assay Protocol

**Figure 3:**
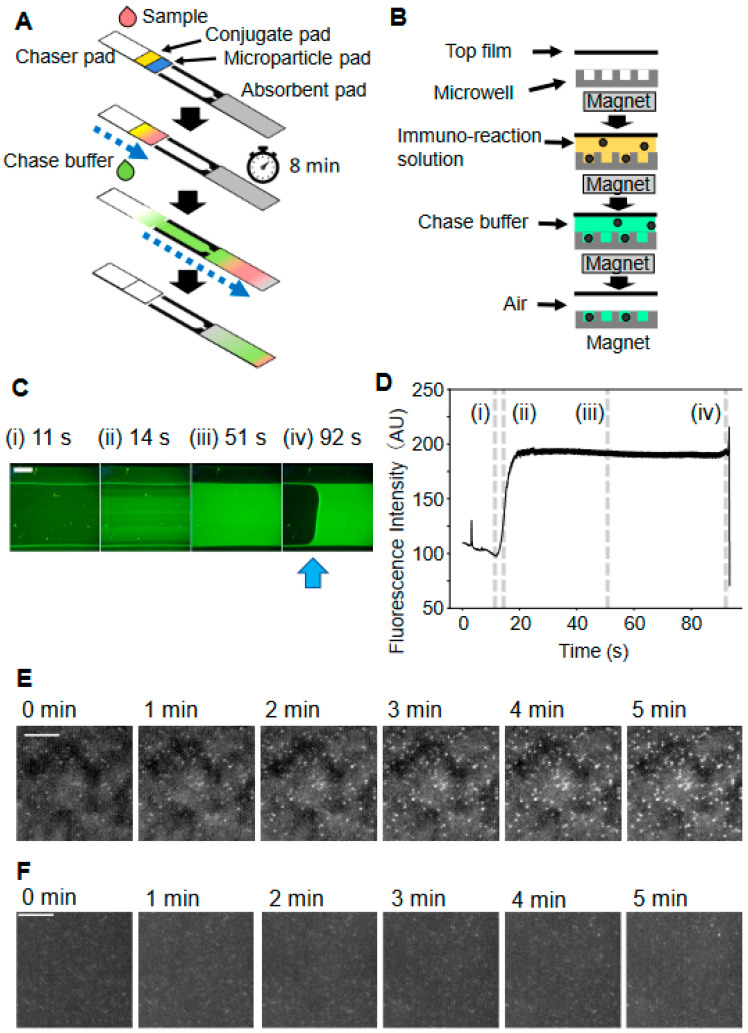
D-strip assay mechanism: (**A**) Schematic of the assay procedure. The sample solution and chase buffer are dropped onto the chase pad sequentially. The flow of the sample solution containing rehydrated reagents and the chase buffer is indicated by the blue dashed line. The lamination film and base plate are omitted from the schematic. Images are not to the scale. (**B**) Schematic of the flow cell cross-section. Chase buffer trails the immuno-reaction solution into the flow cell. Then, magnetic microparticles are trapped in the microwells due to the magnet under the microwell film. The chase buffer washes out the excess immuno-reaction solution in the flow cell. Finally, following the depletion of the chase buffer in the flow cell, each microwell becomes encapsulated by a physical barrier of air (air-sealing). Images are not to the scale. (**C**) Representative sequential images of the flow cell upon blue light irradiation: (**i**) before chase buffer injection, (**ii**) just after the initiation of chase buffer flow, (**iii**) during the chase buffer flow, and (**iv**) depletion of chase buffer. The boundary between the chase buffer and air is indicated by the blue arrow. The time indicates the time elapsed after the injection of the chase buffer. Scale bar: 100 µm. (**D**) Time trajectory of intensity at the center of the image shown in (**C**). (**i**–**iv**) mark with dashed line correspond with the time point in (**C**), respectively. (**E**) Sequential fluorescence images for positive sample (10^5^ copies/mL) at 1 min intervals during the enzyme reaction. The total enzyme reaction time at room temperature was 5 min. The appearance and intensification of fluorescent spots were time-dependent. Scale bar: 100 µm. (**F**) Sequential fluorescence images for the negative sample at 1 min intervals. Scale bar: 100 µm.

To initiate the D-strip assay, the strip tray was manually opened (Figure 2C), and a strip was placed on the tray. A diluted sample (60 µL) was then injected into the chaser pad of the strip on the open tray (Figure 2C and Figure 3A). Once the tray was closed, the system recognized this action and automatically initiated the D-strip assay process. Upon closing the tray, the outlet of the chase buffer from the pump was aligned with the top of the chase pad (Figure 2E). First, the camera lens was auto-focused on the microwells. After an 8 min reaction at room temperature, 210 µL of chase buffer was automatically dispensed via a pump-driven syringe onto the chaser pad. The chase buffer flowed through the chaser pad, reagent pads, and the flow cell and was absorbed by the absorbent pad. A series of flow cell images under blue illumination was recorded until the green fluorescence decreased due to the depletion of the chase buffer (Figure 3C,D). The chase buffer influx was detected by thresholding the mean gray value in a 100-pixel rectangle region of interest (ROI) at the center of the green channel image. Following the depletion of the chase buffer in the flow cell, each microwell was isolated by an air gap (Figure 3B), and enzyme reactions in each well were spontaneously initiated. Therefore, the time when the mean gray value in the flow cell dropped lower than the set threshold value was regarded as the onset of enzyme reaction. Upon the initiation of the enzyme reaction, camera autofocusing on the microwells was performed again. Then, six full-size images of the particles and fluorescence were sequentially captured at 1 min intervals under green and blue illumination, respectively.

### 2.5. Image Processing of Images Obtained from the D-Strip Assay

The original image processing algorithm was developed in a previous study [11] and was partially modified for the current study as follows. A series of six particle and fluorescence images were aligned to the primary image using StackReg [25] to correct slight displacements due to mechanical drift during the 5 min enzyme reaction period. The contrast between particle-occupied and empty microwells was maximized by projecting the minimum value for each pixel of the serial images. Fluorescent spots derived from conjugate activity were highlighted by the slope of the incremental fluorescence intensity at each pixel. The image registration and the object enhancement described above were concurrently performed with sequential image acquisition during enzyme reaction. A Top-Hat filter with a 4- and 20-pixel disk to remove debris and background, respectively, was applied to the particle and fluorescence image pair. Peaks in particle and fluorescence images within 2048 × 2048 pixels ROI were extracted by global thresholding with Gaussian blur and maximum filter. The image processing pipeline was implemented using the Python scikit-image module [26].

Signal%, which is proportional to the concentration of analyte, was calculated based on the number of extracted peaks in the particle and fluorescence images as follows:

$$Signal\%=\frac{The\;number\;of\;fluorescence\;spots\;matched\;to\;particle}{The\;number\;of\;particles}\times 100$$

The number of fluorescent spots spatially matched to a particle was calculated by dilating the binary peak images with a 3 × 3 pixels element, and the union of particle and fluorescence peaks was extracted by a logical AND operation. Objects in the union image greater than 4 pixels in size were counted as a fluorescent spot matched to a particle.

### 2.6. Lateral Flow Assay and Its Analysis

For comparative analysis, a conventional LFA, Panbio^®^ COVID-19 Antigen rapid test (Abbott Laboratories, Chicago, IL, USA), was evaluated according to the package insert instructions [27] with slight modifications. The inactivated virus solution was substituted for a swab specimen for D-strip assay.

The LFA quantification study was performed as follows. All images were aligned with a representative image, and line profiles along the same line ROI were extracted. The line profile background was removed by first-order differentiation. The maximum intensity of the background-subtracted line profile around the test bar was defined as the signal intensity.

## 3. Results

### 3.1. Assay Mechanism

To assemble the D-strip flow cell, the reagent pads, chaser pad, absorbent pad, and top film were adhered to a microwell film via a flow cell-patterned double-sided tape (Figure 1A,B). Then, the assembled strip was mounted on a base plate via a double-sided tape (Figure 1A,C). The assay was performed using the developed D-strip assay platform (Figure 2A,B).

A schematic of the D-strip assay is shown in Figure 3A. An assay was initiated by injecting a sample solution into the chaser pad, which rehydrated the reagents in the pads. Then, the sample solution was diffused to the adjacent conjugate pad. Next, dissolved conjugates migrated along with the sample solution to the downstream particle pad. Following the rehydration of the dried, coated microparticles by the sample solution containing alkaline phosphatase (AP)-labeled antibodies, an immunoreaction between the sample, conjugates, and microparticles occurred on the pad. A polypropylene tape laminate over the reagent pads (Figure 1A) ensured proper liquid flow, directing the applied solution from the chaser pad through the reagent pads to the flow cell, preventing bypass flow. After an immuno-reaction time of 8 min, a chase buffer was applied to the chaser pad, which pushed the immuno-reaction solution into the adjacent flow cell. As the sample solution passed the flow cell, the floating magnetic particles were attracted by the magnet located under the strip, and the particles were trapped in the microwell array (Figure 3B). Excess conjugate and sample solution were washed away by the flow of the chase buffer. The influx of the chase buffer was indicated by fluorescence emission from the pyranine–phosphate present in the chase buffer (Figure 3C,D). As the flow reached the edge of the channel, the sample and then eventually the chase buffer were absorbed by the absorbent pad, decreasing the green fluorescence emission (Figure 3C(iv),D) and indicating the onset of enzyme reactions in the microwells. The process of injection to the depletion of chase buffer took about 1 min (Figure 3C,D). To guarantee efficient liquid absorption from the flow cell to the absorbent pad, Tween-20 was coated on a microwell film between the flow cell outlet and the absorbent pad. In the isolated microwells containing the ternary complex of conjugate, mP) and antigen, pyranine–phosphate in the chase buffer was cleaved into pyranine by AP on the conjugate, producing pyranine’s green fluorescence emission. In microwells without the ternary complex, no green fluorescence was generated. As a result, the microwells filled with chase buffer exhibited a time-dependent increase in bright fluorescent spots as the enzyme reaction progressed in positive samples (Figure 3E), while only a limited number of bright fluorescent spots were observed in negative samples (Figure 3F).

A serial acquisition of particle and fluorescence images (6 images each) during the 5 min enzyme reaction was automatically initiated immediately after detecting the onset of enzyme reaction as described above. Although fluctuations in intensity in the serial particle images were frequently found, the minimum projection at each pixel was effective in retaining the contrast between particle-occupied and empty microwells. Debris in the field of view was masked by image processing, and the peak positions of particles and fluorescent spots were detected, while false positive detections on debris were eliminated by the mask. For digital counting, peak detection beyond a set threshold was used to identify particle positions, while fluorescent spots were detected by peak detection based on the incremental fluorescence intensity slope at each pixel. Finally, the signal intensity was calculated from the number of detected particles and fluorescence spots. All these processes are fully integrated into the assay platform, resulting in a sample-to-result time of 15 min.

### 3.2. Analytical Sensitivity

**Figure 4:**
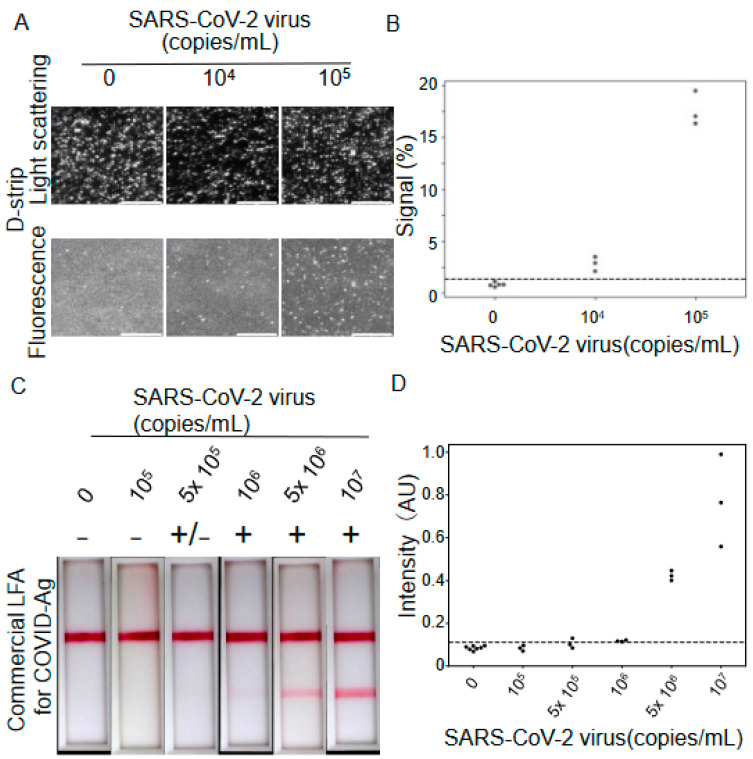
Results of analytical sensitivity studies for the D-strip assay and LFA: (**A**) Representative green light scatter and fluorescence images of the microwell array. The assay was performed using the SARS-CoV-2 panel, as indicated at the top of the images. Scale bar: 100 µm. (**B**) Quantification of signal in D-strip assay using the SARS-CoV-2 panel with 0 (n = 5), 1.0 × 10^4^ (n = 3) and 1.0 × 10^5^ (n = 3) copies/mL. All experiments were performed in a day. The average of the background signal plus three standard deviations of the background was indicated by the black dashed line. (**C**) Representative images of commercial LFA (COVID-19 Antigen rapid test strip). An assay was performed using the SARS-CoV-2 panel, as indicated at the top of the images. The visual detection result is labeled as + for positive and—for negative above each image. (**D**) Quantification of LFA tests, using SARS-CoV-2 panel with 0 (n = 7), 1.0 × 10^5^ (n = 3), 5.0 × 10^5^ (n = 3), 1.0 × 10^6^ (n = 3), 5.0 × 10^6^ (n = 3), 1.0 × 10^7^ (n = 3) copies/mL. The intensity of the test bar was quantified through image processing. The average of the background signal plus three standard deviations of the background is indicated by the black dashed line.

The analytical sensitivity of the D-strip assay to SARS-CoV-2 antigen was evaluated using 0, 1.0 × 10^4^, and 1.0 × 10^5^ copies/mL inactivated virus panels diluted in buffer (Figure 4A,B and Supplementary Table S1). Arithmetic mean ± standard deviation for the negative sample, 10^4^ cp/mL and 10^5^ cp/mL) was 0.77 ± 0.15 (n = 5), 2.60 ± 0.52 (n = 3) and 16.2 ± 1.56 (n = 3), respectively. Figure 4B shows the test results of experiments in each assay. The limit of detection (LOD) was determined by extrapolating the concentration at which the signal becomes equal to the background signal plus three standard deviations of the background. The calculated LOD of the D-strip assay is 1.7 × 10^3^ copies/mL. For comparison, the measured LOD of a commercially available LFA of COVID-19 Antigen rapid test [28,29] was 8.3 × 10^5^ copies/mL (Figure 4C,D). Consistent with the LODs calculated based on the image intensities (Figure 4D), the 0 and 1.0 × 10^5^ copies/mL inactivated virus panels could not be visually detected by the eye (Figure 4C). Here, the LOD for commercial LFA was calculated in the same manner as that for the D-strip assay (Figure 4D). The D-strip exhibited 488-fold higher sensitivity to SARS-CoV-2 antigen compared with conventional LFAs.

## 4. Discussion

In this study, we developed and evaluated a compact digital immunoassay platform that exhibited 488-fold sensitivity to SARS-CoV-2 antigen compared to that for a conventional LFA. Consistent with our LOD of 8.3 × 10^5^ copies/mL for LFAs using buffer-based samples, comparative studies of the commercially available LFAs to SARS-CoV-2 antigen revealed that the typical LOD of LFAs is about 10^6^ copies/mL using clinical samples [28,29,30]. The LOD for a D-strip SARS-CoV-2 antigen assay (1.7 × 10^3^ copies/mL) was less than 10^4^ copies/mL, which is comparable to the LOD of a clinical laboratory test [31]. It also exceeded the sensitivity of the state-of-the-art ultrasensitive LFA to SARS-CoV-2 antigen [8]. LFAs have been widely used as point-of-care tests. However, their limited sensitivity raises concerns about false negatives at the initial phase of viral infection. While molecular tests are highly sensitive, their high reagent costs restrict their widespread use. With a low LOD of 1.7 × 10^3^ copies/mL, the D-strip offers a cost-effective diagnostic solution, bridging the sensitivity gap between LFAs and molecular tests. The highly sensitive D-strip assay produced a result within 15 min after injecting the sample solution into the test strip, like that for conventional LFAs. Since the time-consuming image analysis was performed in parallel with the assay, it had no influence on the sample-to-result time. Therefore, considering its excellent LOD and short sample-to-result time, the D-strip assay can be an accurate and cost-effective confirmatory assay outside clinical laboratory settings.

The developed D-strip assay platform in this study is the first portable device with a fully integrated digital immunoassay, although compact devices merely providing a read-out of digital bioassay signals have been reported [21,23,32]. To achieve a fully integrated compact instrument, (1) instead of traditional pipetting, we exploited capillary force on the strip to induce flow, (2) a chase buffer with multiple roles was used, (3) oil-free isolation of microwells was designed to eliminate complex liquid handling processes required by existing digital immunoassays, and (4) the optical system and paramagnetic particle capture systems were re-designed to reduce the footprint of the assay system.

The flow cell was mainly composed of porous materials commonly used in LFAs, while the nitrocellulose membrane was replaced by a thin flow cell (Figure 1A). Passive flow driven by the strip materials enabled solution mixing and replacement without pipetting (Figure 3A). The chase buffer played three roles in the flow cell: pushing paramagnetic particles from the pad, washing unbound conjugates away, and acting as a substrate solution. This reduced the number of liquid components and is similar to enzymatic signal amplification [2]. Oil-free microwell isolation significantly contributed to minimizing the liquid handling mechanism. Whereas a previous report showed that actively supplied air functioned as a microwell sealant [20], our study revealed that a passive replacement of excess solution with air by a wicking pad could isolate microwells from each other without mechanical actions for sealant injection. The absorption of chase buffer in the flow cell by the absorbent pad on the D-strip is enough to isolate each microwell. In addition, it is noteworthy that the elimination of fluorinated oil removed a major disadvantage of existing digital immunoassays, which is the potential risk of releasing toxic perfluorocarbon into the environment [33]. Lastly, the arrangement of the optical system in our platform ensured that the magnet was not in the field of view during image acquisition (Figure 2B). The previously reported benchtop systems required a mechanism to move the magnet away after seeding magnetic particles into the microwells to acquire microwell array images [11,15]. In contrast, the D-strip assay platform imaged the microwells through a transparent film on top of the flow cell while the magnet was located on the opposite side. These process and design improvements greatly downsized the size and complexity of our immunoassay system. Moreover, the operator actions required are the same as those required by conventional LFAs: injecting sample solution and chase buffer. The D-strip assay platform combined the high sensitivity of digital ELISA with the convenience of LFA.

It is expected that our findings can be potentially applied to other digital bioassays with some modifications. The D-strip assay process is partially compatible with digital nucleic acid detection, digital polymerase chain reaction (PCR) [34], and CRISPR-based diagnostics (CRISRPDx) using microwell arrays [32,35]. The D-strip assay platform enables quantitative and highly sensitive molecular tests on a compact footprint. Microwell-based enzymatic activity profiling at single molecule resolution [36,37] may also be possible, paving the way for cancer and diabetes diagnostic applications.

In this study, all assays were performed at room temperature in a laboratory. To determine the feasibility of the D-strip assay platform for PoCT, it should be applied under different uncontrolled environments out of the laboratory, and its performance in accurately assessing nasopharyngeal swabs and saliva specimens for different diseases should be comprehensively evaluated.

In conclusion, the D-strip combines the advantages of digital ELISA and LFA, providing a very sensitive immunoassay platform that is portable, cost-effective, simple, and accurate.

## 5. Patents

The authors declare that the content of this manuscript has been filed as a patent by Abbott Laboratories.

## Data Availability

Dataset available on request from the corresponding author.

## Supplementary Materials

The following supporting information can be downloaded at: https://www.mdpi.com/article/10.3390/biomedicines12112517/s1, Table S1: Mean signal percentages of D-strip assay using SARS-CoV-2 panel.

## References

[1] Mitamura K., Shimizu H., Yamazaki M., Ichikawa M., Nagai K., Katada J., Wada A., Kawakami C., Sugaya N. (2013). Clinical evaluation of highly sensitive silver amplification immunochromatography systems for rapid diagnosis of influenza. J. Virol. Methods.

[2] Panferov V.G., Safenkova I.V., Varitsev Y.A., Zherdev A.V., Dzantiev B.B. (2017). Enhancement of lateral flow immunoassay by alkaline phosphatase: A simple and highly sensitive test for potato virus X. Microchim. Acta.

[3] Kim C., Yoo Y.K., Han S.I., Lee J., Lee D., Lee K., Hwang K.S., Lee K.H., Chung S., Lee J.H. (2017). Battery operated preconcentration-assisted lateral flow assay. Lab. Chip.

[4] Sharma A., Tok A.I.Y., Lee C., Ganapathy R., Alagappan P., Liedberg B. (2019). Magnetic Field Assisted Preconcentration of Biomolecules for Lateral Flow Assaying. Sens. Actuators B Chem..

[5] Katis I.N., He P.J.W., Eason R.W., Sones C.L. (2018). Improved sensitivity and limit-of-detection of lateral flow devices using spatial constrictions of the flow-path. Biosens. Bioelectron..

[6] Park S.B., Shin J.H. (2022). Pressed Lateral Flow Assay Strips for Flow Delay-Induced Signal Enhancement in Lateral Flow Assay Strips. Biochip J..

[7] Sena-Torralba A., Torné-Morató H., Parolo C., Ranjbar S., Nejad M.A.F., Álvarez-Diduk R., Idili A., Hormozi-Nezhad M.R., Merkoçi A. (2022). A Novel Ratiometric Fluorescent Approach for the Modulation of the Dynamic Range of Lateral Flow Immunoassays. Adv. Mater. Technol..

[8] Gupta R., Gupta P., Wang S., Melnykov A., Jiang Q., Seth A., Wang Z., Morrissey J.J., George I., Gandra S. (2023). Ultrasensitive lateral-flow assays via plasmonically active antibody-conjugated fluorescent nanoparticles. Nat. Biomed. Eng..

[9] Rissin D.M., Kan C.W., Campbell T.G., Howes S.C., Fournier D.R., Song L., Piech T., Patel P.P., Chang L., Rivnak A.J. (2010). Single-molecule enzyme-linked immunosorbent assay detects serum proteins at subfemtomolar concentrations. Nat. Biotechnol..

[10] Kim S.H., Iwai S., Araki S., Sakakihara S., Iino R., Noji H. (2012). Large-scale femtoliter droplet array for digital counting of single biomolecules. Lab. Chip.

[11] Chiba R., Miyakawa K., Aoki K., Morikawa T.J., Moriizumi Y., Degawa T., Arai Y., Segawa O., Tanaka K., Tajima H. (2022). Development of a Fully Automated Desktop Analyzer and Ultrahigh Sensitivity Digital Immunoassay for SARS-CoV-2 Nucleocapsid Antigen Detection. Biomedicines.

[12] Kuzmichev Y.V., Lackman-Smith C., Bakkour S., Wiegand A., Bale M.J., Musick A., Bernstein W., Aronson N., Ake J., Tovanabutra S. (2023). Application of ultrasensitive digital ELISA for p24 enables improved evaluation of HIV-1 reservoir diversity and growth kinetics in viral outgrowth assays. Sci. Rep..

[13] Arai Y., Wang D., Takeuchi M., Utsunomiya S., Degawa T., Kai A., Ichikawa H., Chiba R., Yoshimura T. (2023). Development of a quantitative thyroid-stimulating hormone assay system for a benchtop digital ELISA desktop analyzer. Front. Bioeng. Biotechnol..

[14] Suzuki N., Takeuchi M., Miyazaki N., Tanaka K., Utsunomiya S., Arai Y., Yoshimura T., Sugino K., Ito K., Noh J.Y. (2024). Determination of Capillary Blood TSH and Free Thyroxine Levels Using Digital Immunoassay. J. Endocr. Soc..

[15] Leirs K., Dal Dosso F., Perez-Ruiz E., Decrop D., Cops R., Huff J., Hayden M., Collier N., Yu K.X.Z., Brown S. (2022). Bridging the Gap between Digital Assays and Point-of-Care Testing: Automated, Low Cost, and Ultrasensitive Detection of Thyroid Stimulating Hormone. Anal. Chem..

[16] Wilson D.H., Rissin D.M., Kan C.W., Fournier D.R., Piech T., Campbell T.G., Meyer R.E., Fishburn M.W., Cabrera C., Patel P.P. (2016). The Simoa HD-1 Analyzer: A Novel Fully Automated Digital Immunoassay Analyzer with Single-Molecule Sensitivity and Multiplexing. J. Lab. Autom..

[17] Wang T., Zhang M., Dreher D.D., Zeng Y. (2013). Ultrasensitive microfluidic solid-phase ELISA using an actuatable microwell-patterned PDMS chip. Lab. Chip.

[18] Rondelez Y., Tresset G., Tabata K.V., Arata H., Fujita H., Takeuchi S., Noji H. (2005). Microfabricated arrays of femtoliter chambers allow single molecule enzymology. Nat. Biotechnol..

[19] Yaginuma H., Ohtake K., Akamatsu T., Noji H., Tabata K.V. (2022). A microreactor sealing method using adhesive tape for digital bioassays. Lab. Chip.

[20] Honda S., Minagawa Y., Noji H., Tabata K.V. (2021). Multidimensional Digital Bioassay Platform Based on an Air-Sealed Femtoliter Reactor Array Device. Anal. Chem..

[21] Yelleswarapu V., Buser J.R., Haber M., Baron J., Inapuri E., Issadore D. (2019). Mobile platform for rapid sub-picogram-per-milliliter, multiplexed, digital droplet detection of proteins. Proc. Natl. Acad. Sci. USA.

[22] Minagawa Y., Ueno H., Tabata K.V., Noji H. (2019). Mobile imaging platform for digital influenza virus counting. Lab. Chip.

[23] Chen H., Li Z., Zhang L., Sawaya P., Shi J., Wang P. (2019). Quantitation of Femtomolar-Level Protein Biomarkers Using a Simple Microbubbling Digital Assay and Bright-Field Smartphone Imaging. Angew. Chem..

[24] Yamaoka Y., Miyakawa K., Jeremiah S.S., Funabashi R., Okudela K., Kikuchi S., Katada J., Wada A., Takei T., Nishi M. (2021). Highly specific monoclonal antibodies and epitope identification against SARS-CoV-2 nucleocapsid protein for antigen detection tests. Cell Rep. Med..

[25] Thevenaz P., Ruttimann U.E., Unser M. (1998). A pyramid approach to subpixel registration based on intensity. IEEE Trans. Image Process..

[26] van der Walt S., Schonberger J.L., Nunez-Iglesias J., Boulogne F., Warner J.D., Yager N., Gouillart E., Yu T., The Scikit-Image Contributors (2014). Scikit-image: Image processing in Python. PeerJ.

[27] (2021). Abbott Laboratories Panbio COVID-19 Ag Rapid Test, Package Insert. https://dam.abbott.com/en-gb/panbio/120007883-v1-Panbio-COVID-19-Ag-Nasal-AsymptomaticSe.pdf.

[28] Corman V.M., Haage V.C., Bleicker T., Schmidt M.L., Muhlemann B., Zuchowski M., Jo W.K., Tscheak P., Moncke-Buchner E., Muller M.A. (2021). Comparison of seven commercial SARS-CoV-2 rapid point-of-care antigen tests: A single-centre laboratory evaluation study. Lancet Microbe.

[29] Cubas-Atienzar A.I., Kontogianni K., Edwards T., Wooding D., Buist K., Thompson C.R., Williams C.T., Patterson E.I., Hughes G.L., Baldwin L. (2021). Limit of detection in different matrices of 19 commercially available rapid antigen tests for the detection of SARS-CoV-2. Sci. Rep..

[30] Pickering S., Batra R., Merrick B., Snell L.B., Nebbia G., Douthwaite S., Reid F., Patel A., Kia Ik M.T., Patel B. (2021). Comparative performance of SARS-CoV-2 lateral flow antigen tests and association with detection of infectious virus in clinical specimens: A single-centre laboratory evaluation study. Lancet Microbe.

[31] Menchinelli G., Bordi L., Liotti F.M., Palucci I., Capobianchi M.R., Sberna G., Lalle E., Romano L., De Angelis G., Marchetti S. (2021). Lumipulse G SARS-CoV-2 Ag assay evaluation using clinical samples from different testing groups. Clin. Chem. Lab. Med..

[32] Iida T., Ando J., Shinoda H., Makino A., Yoshimura M., Murai K., Mori M., Takeuchi H., Noda T., Nishimasu H. (2023). Compact wide-field femtoliter-chamber imaging system for high-speed and accurate digital bioanalysis. Lab. Chip.

[33] Lohmann R., Cousins I.T., DeWitt J.C., Gluge J., Goldenman G., Herzke D., Lindstrom A.B., Miller M.F., Ng C.A., Patton S. (2020). Are Fluoropolymers Really of Low Concern for Human and Environmental Health and Separate from Other PFAS?. Environ. Sci. Technol..

[34] Men Y., Fu Y., Chen Z., Sims P.A., Greenleaf W.J., Huang Y. (2012). Digital polymerase chain reaction in an array of femtoliter polydimethylsiloxane microreactors. Anal. Chem..

[35] Shinoda H., Taguchi Y., Nakagawa R., Makino A., Okazaki S., Nakano M., Muramoto Y., Takahashi C., Takahashi I., Ando J. (2021). Amplification-free RNA detection with CRISPR-Cas13. Commun. Biol..

[36] Sakamoto S., Komatsu T., Watanabe R., Zhang Y., Inoue T., Kawaguchi M., Nakagawa H., Ueno T., Okusaka T., Honda K. (2020). Multiplexed single-molecule enzyme activity analysis for counting disease-related proteins in biological samples. Sci. Adv..

[37] Nagano N., Ichihashi Y., Komatsu T., Matsuzaki H., Hata K., Watanabe T., Misawa Y., Suzuki M., Sakamoto S., Kagami Y. (2023). Development of fluorogenic substrates for colorectal tumor-related neuropeptidases for activity-based diagnosis. Chem. Sci..

