# Supplementary figures and images for: Compact Digital Immunoassay Platform Integrating ELISA with a Lateral Flow Strip
Source: Biomedicines. 2024 Nov 4;12(11):2517. doi: 10.3390/biomedicines12112517 (PMC11591789; doi:10.3390/biomedicines12112517)

## Slide 1
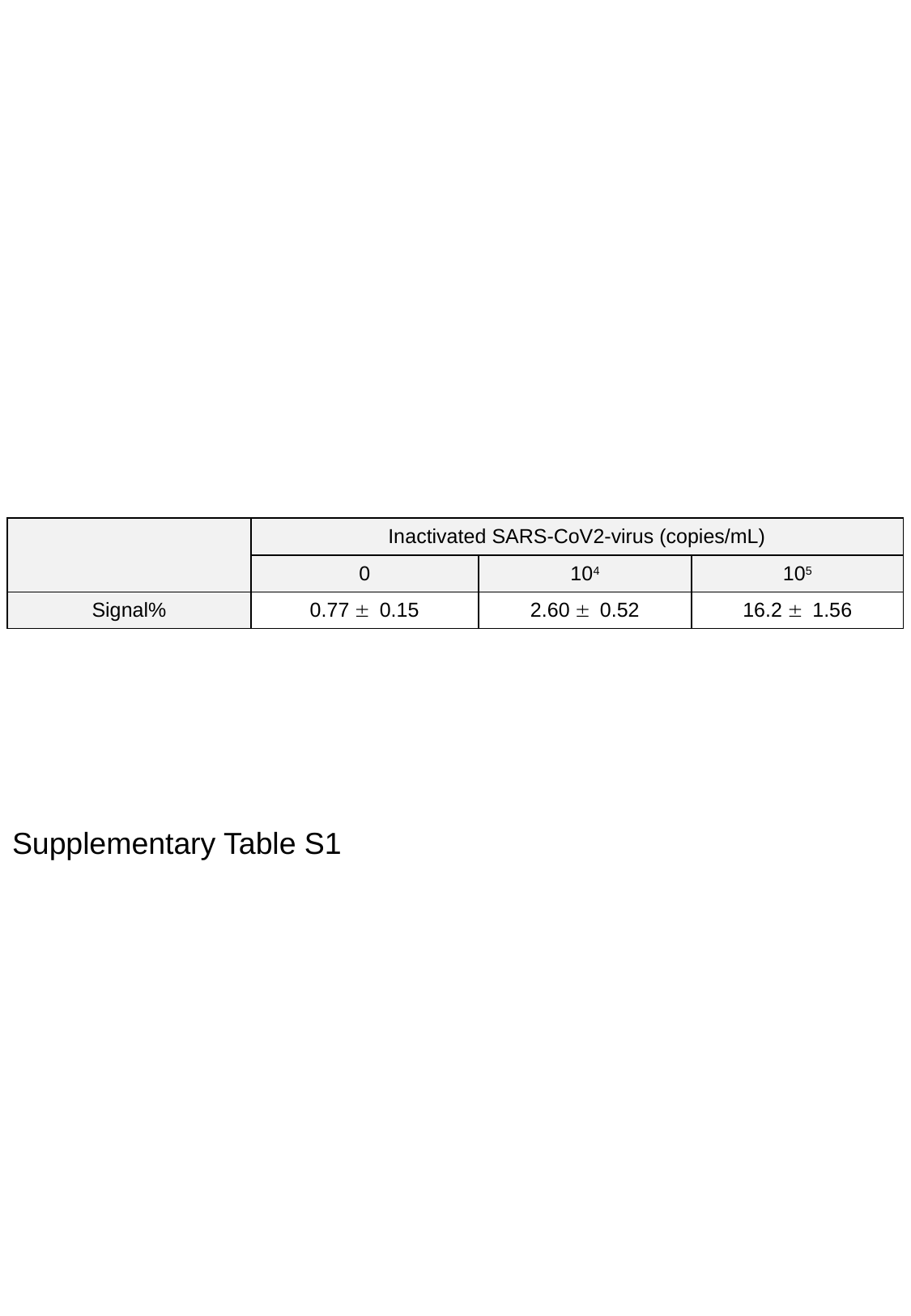

| | Inactivated SARS-CoV2-virus (copies/mL) | | |
| --- | --- | --- | --- |
| | 0 | 104 | 105 |
| Signal% | 0.77 ± 0.15 | 2.60 ± 0.52 | 16.2 ± 1.56 |
Supplementary Table S1

Supplement: Supplementary file 1 [file biomedicines-12-02517-s001.zip › biomedicines-3261147-supplementary.pptx]
